# Emotion recognition based on EEG features in movie clips with channel selection

**DOI:** 10.1007/s40708-017-0069-3

**Published:** 2017-07-15

**Authors:** Mehmet Siraç Özerdem, Hasan Polat

**Affiliations:** 1 Electrical and Electronics Engineering, Dicle University, 21000 Diyarbakır, Turkey; 2 Electrical and Electronics Engineering, Mus Alparslan University, 49000 Muş, Turkey

**Keywords:** Emotion, EEG, Classification, Wavelet transform, Channel selection

## Abstract

Emotion plays an important role in human interaction. People can explain their emotions in terms of word, voice intonation, facial expression, and body language. However, brain–computer interface (BCI) systems have not reached the desired level to interpret emotions. Automatic emotion recognition based on BCI systems has been a topic of great research in the last few decades. Electroencephalogram (EEG) signals are one of the most crucial resources for these systems. The main advantage of using EEG signals is that it reflects real emotion and can easily be processed by computer systems. In this study, EEG signals related to positive and negative emotions have been classified with preprocessing of channel selection. Self-Assessment Manikins was used to determine emotional states. We have employed discrete wavelet transform and machine learning techniques such as multilayer perceptron neural network (MLPNN) and *k*-nearest neighborhood (*k*NN) algorithm to classify EEG signals. The classifier algorithms were initially used for channel selection. EEG channels for each participant were evaluated separately, and five EEG channels that offered the best classification performance were determined. Thus, final feature vectors were obtained by combining the features of EEG segments belonging to these channels. The final feature vectors with related positive and negative emotions were classified separately using MLPNN and *k*NN algorithms. The classification performance obtained with both the algorithms are computed and compared. The average overall accuracies were obtained as 77.14 and 72.92% by using MLPNN and *k*NN, respectively.

## Introduction

Emotion is a human consciousness and plays a critical role in rational decision-making, perception, human interaction, and human intelligence. While emotions can be reflected through non-physiological signals such as words, voice intonation, facial expression, and body language, many studies on emotion recognition based on these non-physiological signals have been reported in recent decades [1, 2].

Signals obtained by recording voltage changes occurring on skull surface as a result of electrical activity of active neurons in the brain are called EEG [3]. From the clinical point of view, EEG is the mostly used brain-activity-measuring technique for emotion recognition. Furthermore EEG-based BCI systems provide a new communication channel by detecting the variation in the underlying pattern of brain activities while performing different tasks [4]. However, BCI systems have not reached the desired level to interpret people’s emotions.

The interpretation of people’s different emotional states via BCI systems and automatic identification of the emotions may enable robotic systems to emotionally react to humans in the future. They will be more useful especially in fields such as medicine, entertainment, education and in many other areas [5]. BCI systems need variable resources that can be taken from humans and processed to understand emotions. EEG signal is one of the most important resources to achieve this target. Emotion recognition is combined with different areas of knowledge such as psychology, neurology and engineering. SAM questionnaires are usually used for classified affective responses of subjects in the design of emotion recognition systems [6]. However, affective responses are not easily classified into distinctive emotion responses due to the overlapping of emotions.

Emotions can be discriminated with either discrete classification spaces or dimensional spaces. A discrete space allows the assessment of a few basic emotions such as happiness and sadness and is more suitable for unimodal systems [7]. A dimensional space (valence–arousal plane) allows a continuous representation of emotions on two axes. Valence dimension is ranging from unpleasant to pleasant, and arousal dimension is ranging from calm to excited state [8]. Higher dimension is better in the understanding of different states, but the classification accuracies of application can be lower as obtained in Ref [7]. Thus, in this study, EEG signals that are related to positive and negative emotions have been classified with channel selection for only valence dimension.

In the literature, there are studies in which various signals obtained/measured from people are used in order to determine emotions automatically. We can gather these studies under three areas [9]. The first approach includes studies intended to predict emotions using face expressions and/or speech signals [10]. However, the main disadvantage of this approach is that permanently catching the spontaneous face expressions that do not reflect real emotions is quite difficult. Speech and facial expressions vary across cultures and nations as well. The second main approach is based on emotion prediction by tracking the changes in central automatic nervous system [11, 12]. Various signals such as electrocardiogram (ECG), skin conductance response (SCR), breath rate, and pulse are recorded; hence, emotion recognition is applied by processing them. The third approach includes studies intended for EEG-based emotion recognition.

In order to recognize emotions, a large variety of studies were specifically conducted within the scope of EEG signals. These studies can simply be gathered under three main areas; health, game, and advertisement. Studies in health are generally conducted by physicians for purposes of helping in disease diagnosis [13–15]. Game sector involve studies in which people use EEG recordings instead of joysticks and keyboards [16, 17]. As per this study, advertisement sector generally involves studies which aim at recognizing emotions from EEG signals.

There are several studies in which different algorithms related to EEG-based classification of emotional states were used. Some main studies related with emotions are given below.

- Murugappan et al. [18] classified five emotions based on EEG signals. They used EEG signals that recorded from 64, 24, and 8 EEG channels, respectively. They achieved maximum classification accuracy of 83.26 and 75.21% using *k*NN and linear discriminant analysis (LDA) algorithm, respectively. Researchers employed DWT method for decomposing the EEG signal into alpha, beta, and gamma bands. These frequency bands were analyzed for feature extraction.
- Channel et al. [19] classified two emotions. They employed SAM to determine participant emotions. They used Naive Bayes (NB) and Fisher discriminant analysis (FDA) as the classification algorithms. Classification accuracy was obtained as 72 and 70% for NB and FDA, respectively.
- Zhang et al. [20] applied PCA method for feature extraction. The features were extracted from two channels (F3 and F4). The classification accuracy was obtained as 73% by the researchers.
- Bhardwaj et al. [21] recognized seven emotions using support vector machine (SVM) and LDA. Three EEG channels (Fp1, P3 and O1) were used in their experiment. Researchers investigated sub-bands (theta, alpha and beta) of EEG signal. The overall average accuracies obtained were 74.13% using SVM and 66.50% using LDA.
- Lee et al. [22] classified positive and negative emotions. Classification accuracy was obtained as 78.45% by using adaptive neuro-fuzzy inference system (ANFIS).

In reference to the literature, it is seen that a limited number of EEG channels (e.g., two or three) have been used to detect different emotional states with different classification algorithms such as SVM, MLPNN, and *k*NN.

**Fig. 1:**
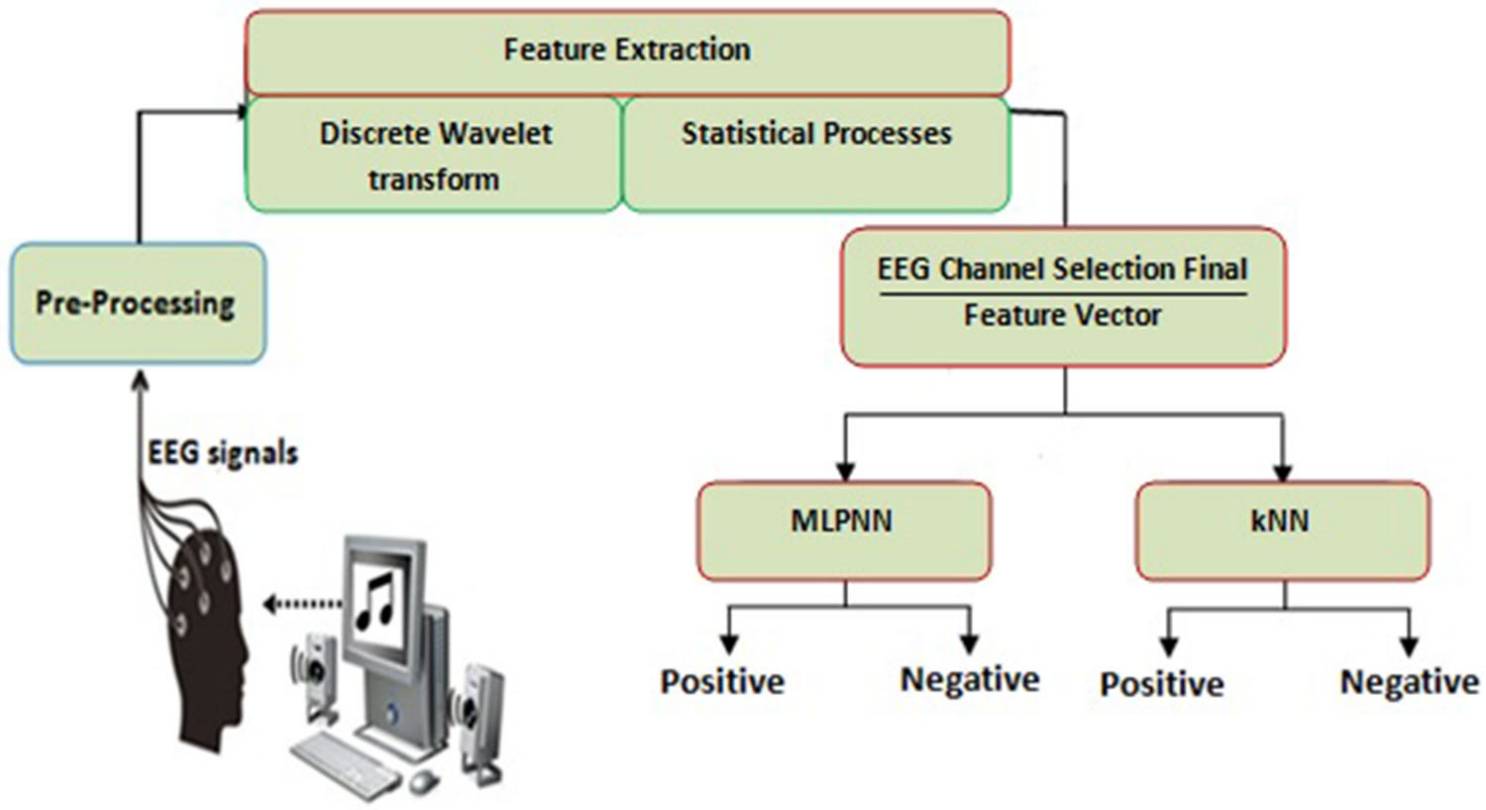
Proposed classification process

The aim of this study was to classify EEG signals related to different emotions based on audiovisual stimuli with the preprocessing of channel selection. SAM was used to determine participants’ emotional states. Participants rated each audiovisual stimulus in terms of the level of valence, arousal, like/dislike, dominance and familiarity. EEG signals that related to positive and negative emotions have been classified according to participants’ valence ratings. DWT method was used for feature extraction from EEG signals. Wavelet coefficients of EEG signals were assumed as feature vectors and statistical features were used to reduce the dimension of those feature vectors. EEG signals related to positive and negative emotions groups have been classified by MLPNN and *k*NN algorithm. After the preprocessing and feature extraction stages, classifiers were used for channel selection (Fig. 1). EEG channels that offer the best classification performance were determined. Thus, final feature vectors were obtained by combining the features of those EEG channel. The final feature vectors were classified and their performances were compared. The steps followed in the classification process are depicted in Fig. 1.

As a remainder, this paper is organized as follows Sects. 2 and 3 describe the Materials and Methods employed in the proposed EEG-based emotion recognition system. Section 4 presents the experimental results. Section 5 presents the results and discussion. Finally, Sect. 6 provides the conclusion of this paper.

## Materials

### Database and participants questionnaire

In this study, the publicly available DEAP database related to emotional states was used. The DEAP database contains EEG and peripheral physiological signals of 32 healthy subject [23]. Thirty-two healthy participant (15 female and 17 male), aged between 23 and 37, participated in the experiment. The experiments were performed in Twente and Geneva university laboratories. Participants 1–22 were analyzed in Twente and the remaining ones 23–32 in Geneva. Due to some minor differences between the two universities, participants taken from Twente University were examined in this study.

### Stimulus material

The DEAP database was recorded by using music clips to stimuli emotions in the participants. The music clips used in the experiment were selected in several steps. Initially, the authors selected 120 music clips. Half of these stimuli were selected by semi automatically and another half was selected manually [24]. From the initial collection of 120 music clips, the final 40 test music clips were determined to present in the paradigm. These music clips were selected to elicit emotion prominently. A 1-min segment related to the maximum emotional content was extracted from each music clips, and these segments were presented in final experiment.

### Task

The EEG signals were recorded from each participant in a particular paradigm framework based on audiovisual stimuli. The paradigm consists of two sections and each section contains 20 trials, each consisting of the following steps:

1. Trial number: A 2-s screen displaying the current trial.
2. Fixation: A 5-s fixation crosses.
3. Music Clip: The 60-s display of the music clips to stimuli different emotions.
4. Assessment: SAM employed for valence, arousal, dominance, likes, and familiarity.

**Fig. 2:**
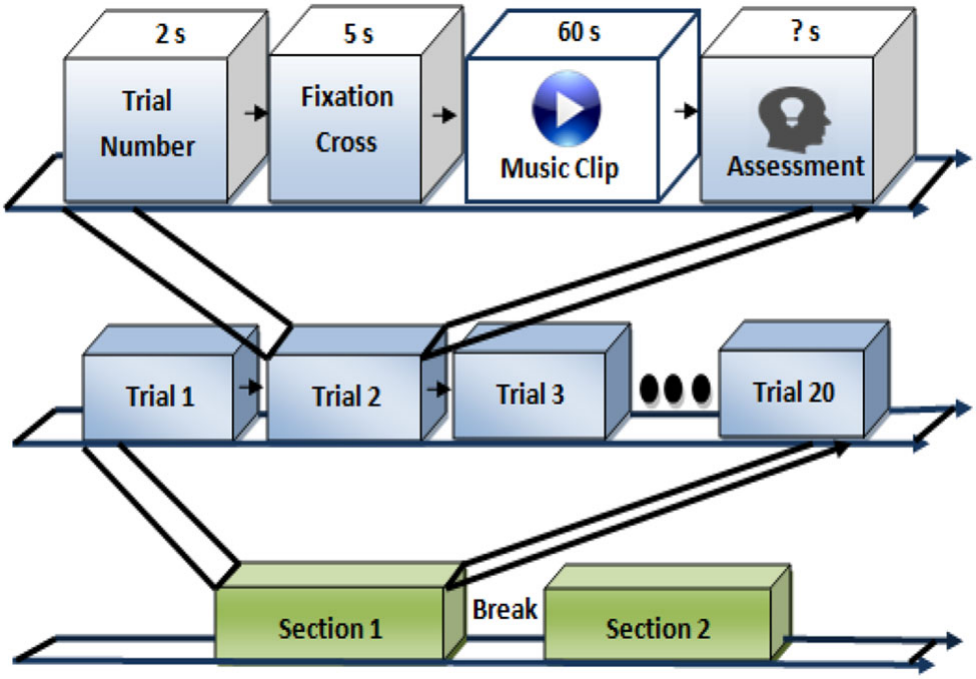
Paradigm applied to record EEG signals from participants

The paradigm applied to participant in order to record EEG and peripheral physiological signals is shown in Fig. 2.

### Participants ratings

**Fig. 3:**
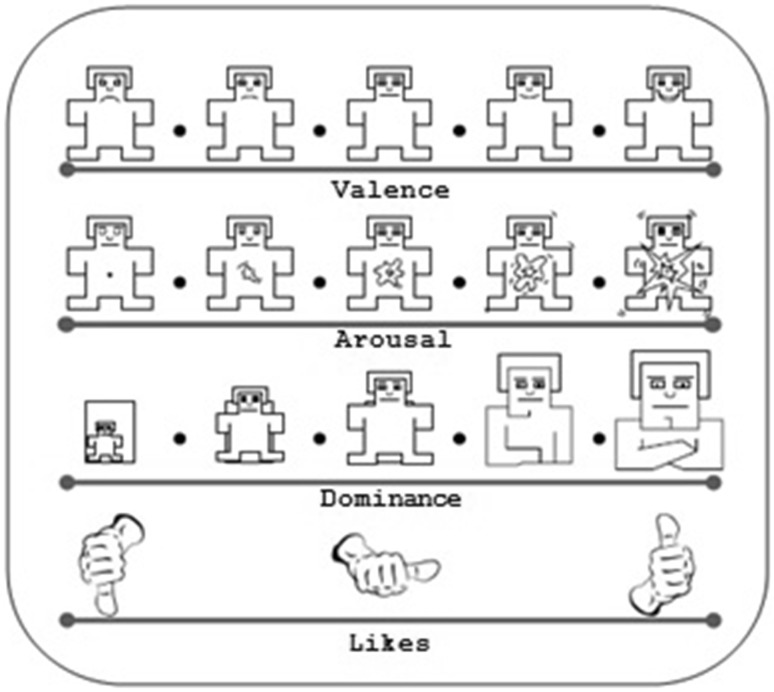
SAM and thumbs-*down*/thumbs-*up* icons

At the end of each music clip, participants assessed their emotional states in terms of valence, arousal, dominance, likes, and familiarity. SAM [25] were used to visualize for valence, arousal and dominance scales. For the likes scale, thumbs-down/thumbs-up icons were used (Fig. 3). Participants assessed each music clip for familiarity effects as well. Participants rated valence, arousal, dominance and likes on a continuous 9-point scale, but the familiarity was rated on a 5-point integer scale. In this study, to recognize the EEG signals related to positive and negative emotions, participants’ evaluations from SAM visuals according to valence panel were taken into consideration. The valence scale is ranging from unhappy or sad to happy or joyful (Fig. 3). The valence ratings of participants were smaller than 5, and this was accepted as negative emotion; else ratings were accepted as positive emotion.

**Fig. 4:**
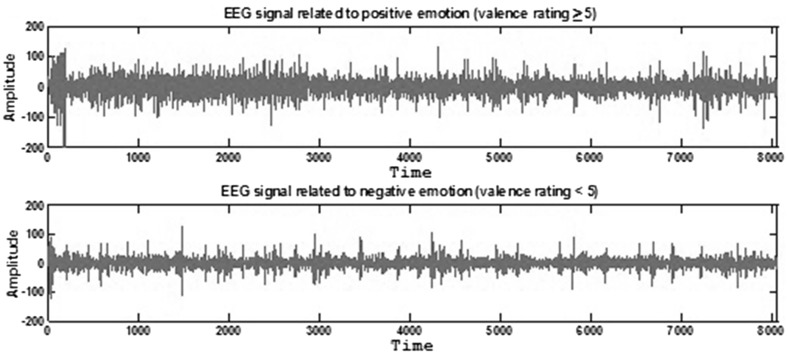
Sample EEG signals related to positive and negative emotions. Sample EEG signals were measured from channel Fp1. *Time axis* was defined as second; *amplitude axis* was defined as µV

In Fig. 4, sample EEG signals related to positive and negative emotions state measured from a randomly selected channel are shown.

### EEG signal recordings

EEG signals related to audiovisual stimuli were recorded using a Biosemi Active Two system. For each participant, EEG signals were recorded from 32 channels. Thirty-two active AgCl electrodes placed according to international 10–20 electrode placement system were used in EEG recordings. EEG signals were digitized by 24-bit analog–digital convertor with 512 Hz sample rate [24].Then, the digitized EEG signals were down sampled to 128 Hz. A band-pass frequency filter from 4.0–45.0 Hz was applied to EEG recordings and electrooculography (EOG) artifacts were removed. Sections that were present before and after the audiovisual stimulus were removed from the recorded EEG signals. Therefore, EEG segment for each music clip were obtained. At the end of these procedures, 40 EEG segments related to 40 music clips for every participant were obtained.

## Methods

In this study, EEG signals related to different emotional states were classified. DWT was used for feature extraction from EEG signals. Wavelet coefficients of EEG signals were assumed as feature vectors and statistical features were used to reduce dimension of those feature vectors.

### Discrete wavelet transform

DWT is widely used for analyzing non-stationary signals [3, 9]. DWT have been famously preferred for analyzing EEG signals. The desired frequency range can be obtained by using DWT method. DWT method decomposes the signal into sub-bands by filtering with consecutive high pass filter *g*[*n*] and low pass filter *h*[*n*] in the time period [10].As a result, first-level *D*1 detail and *A*1 approximation sub-bands of signal are obtained. First-level approximation and detail sub-bands are defined as

1 $$A_{1} = x\left[ n \right]*h\left[ n \right] = \mathop \sum \limits_{k = - \infty }^{\infty } x\left[ k \right] \cdot h\left[ {2n - k} \right]$$

2 $$D_{1} = x\left[ n \right]*g\left[ n \right] = \mathop \sum \limits_{k = - \infty }^{\infty } x\left[ k \right] \cdot g\left[ {2n - k} \right].$$

**Fig. 5:**
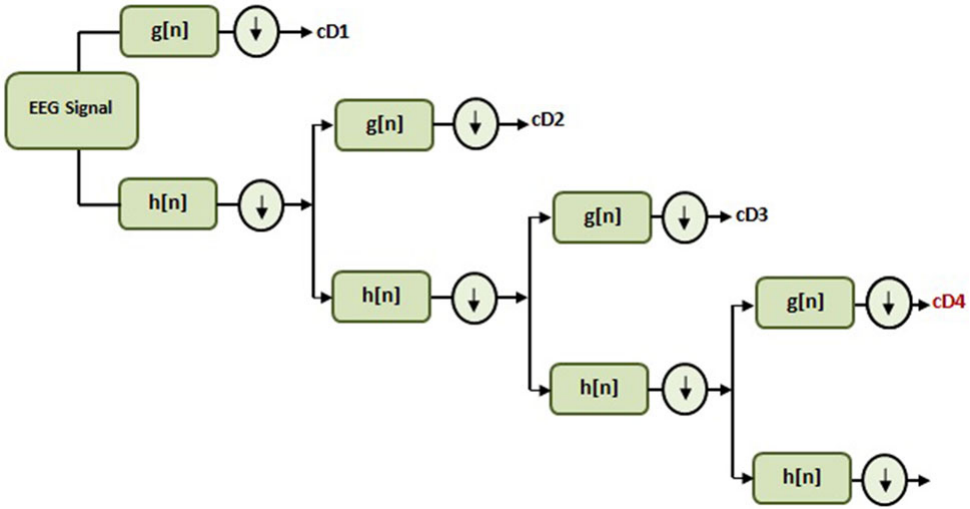
Multi-level signal decomposition by DWT, *h*[*n*] is the low pass and *g*[*n*] is the high pass filter

In order to reach the desired band range, *A*1 called approximation band is re-separated, and procedures are continued consecutively until the intended frequency range is reached. In Fig. 5, decomposition of a EEG signal (*x*[*n*]) into its multi-level frequency ranges via DWT method is shown. Frequency ranges related to detail wavelet coefficients are listed in Table 1 as well.

**Table 1:** Frequency ranges related to detail wavelet coefficients with sampling frequency 128 Hz

Wavelet coefficients	Frequency ranges (Hz)
cD1	32–64
cD2	16–32
cD3	8–16
cD4	4–8 (*θ*)

In literature, EEG theta band dynamics including important information about emotions can be seen [24, 26, 27]. Therefore, theta band dynamics were used in order to obtain feature vectors. Selection of suitable mother wavelet and the number of decomposition levels is very important to analyze non-stationary signals [10]. In this study, the number of decomposition levels was chosen as 4 because theta band dynamics of EEG signals were considered to recognize different emotions based on audiovisual stimulus. Dabuechies wavelets have provided useful results in analyzing EEG signals [10], and hence Daubechies wavelet of order 2 (db2) was chosen in this study.

### Feature extraction

In this study, feature vectors for EEG signals were obtained by using DWT method. The decomposition of the signal creates a set of wavelet coefficients in each sub-band. Theta band wavelet coefficients (cD4) which are composed of 506 samples were assumed as feature vectors. The wavelet coefficients provide a compact representation of EEG features [10]. In order to decrease the dimensionality of feature vector, five statistical parameters were used. The following statistical features were used to reduce dimension of the feature vectors based on theta band;

1. Maximum of the absolute values of the wavelet coefficients.
2. Mean of the absolute values of the wavelet coefficients.
3. Standart deviation of the coefficients.
4. Average power of the coefficients.
5. Average energy of the coefficients.

In this study, above statistical parameters in feature extraction from EEG segments for different emotional states were selected. In addition to these features, different attributes were used. Different combinations have been tried for obtaining the highest success rate. After the calculations, the optimal results were observed by above features.

At the end of DWT method and statistical procedures used for feature extraction, five-dimensional feature vectors belonging to EEG segments related to every emotional state were obtained.

### Classification algorithms

The feature vectors of EEG signals related to positive and negative emotions were classified by MLPNN and *k*NN algorithm. The performances of these algorithms applied for pattern recognition were evaluated and also compared.

#### Artificial neural network

Artificial neural network (ANN) is widely used in engineering area after the development of computer technology [28]. ANN is a computing system inspired from biological neural networks. It consists of an input layer, one or more hidden layer(s), and a output layer. A neural network can be generated by a series of neurons that are contained in these layers. Each neuron in a layer connects with another neuron in the next layer via weights. The weights *w*_*ij*_ represent the connection between the *i*th neuron in a layer and the *j*th neuron in the next layer [29]. Feature vectors obtained after the feature extraction are applied to input layer. All the input components are distributed to the hidden layer. The function of hidden layer is to intervene between the external input and the neurons in the network output. The hidden layer helps to detect nonlinearity in the feature vectors. The set of output signals of the neurons in output layer constitutes the overall response of the network [30, 31]. The neurons of hidden layer process external input. External input is defined as x_*i*_. The first, weighted sum is calculated, and a bias term $\theta_{j}$ is added in order to determine net_*j*_ that is defined as [29]

3 $${\text{net}}_{j} = \mathop \sum \limits_{i = 1}^{m} x_{i} \times w_{ij} + \theta_{j} \quad \left( {j = 1,2, \ldots n} \right)$$

where *m* and *n* are the number of neurons in the input layer and the number neurons of hidden layer, respectively.

In order to transform the net_*j*_ to neurons and in the next layer and generated desired output, a suitable transfer function should be chosen. There are various transfer functions, but the most widely used is the sigmoid one that is defined as [29]

4 $$f\left( x \right) = \frac{1}{{1 + e^{ - x} }}.$$

In this study, a feed-forward back-propagation multilayer neural network was used. The back-propagation training algorithm technique adjusts the weights to obtain network that is closed to the desired output [32].

#### k-nearest neighborhood

*k*NN is a classification algorithm based on distance. *k*NN allows an object encountered in (*n*) dimensional feature space to be assigned to a predetermined class by examining the attributes of a new object [33].In *k*NN algorithm, classification process starts with the calculation of the distance of a new object with unknown class to each object in the training set. Classification process is completed by assigning the new object to the class most common among its *k*-nearest neighbors. Known classes of objects in the training set of *k*-nearest neighbors were taken into account for classification.

Different methods used to calculate the distance in *k*NN algorithm. For these methods, Minkowski, Manhattan, Euclidean, and Hamming distance measures can be given as examples. In this study, the Euclidean distance was used to determine nearest neighbors. The Euclidean distance is defined as

5 $$d = \sqrt {\mathop \sum \limits_{i = 1}^{n} \left( {x_{i} - y_{i} } \right)^{2} }$$

where *x* and *y* are the position of points in a Euclidean *n*-space.

The choice of *k* is optimized by calculating the classification algorithm ability with different *k* values. In order to acquire the best classification accuracy, appropriate value of *k* was determined as different for each participant.

### Performance criteria

Accuracy, specificity, and sensitivity can be used to test the performance of the trained network. These criteria are among the success evaluation criteria which are frequently used in the literature. In this study, specificity represents the ability to correctly classify the samples belonging to a positive emotion; sensitivity represents the ability to correctly classify the samples belonging to a negative emotion. Accuracy, specificity, and sensitivity are computed by Eqs. (6), (7), and (8), respectively.

6 $${\text{Accuracy}} = \frac{{{\text{TP}} + {\text{TN}}}}{{{\text{TP}} + {\text{FP}} + {\text{TN}} + {\text{FN}}}} \times 100\%$$

7 $${\text{Specificity}} = \frac{\text{TP}}{{{\text{TP}} + {\text{FP}}}} \times 100\%$$

8 $${\text{Sensitivity}} = \frac{\text{TN}}{{{\text{TN}} + {\text{FN}}}} \times 100\% .$$

True positive (TP), the number of true decision related to positive emotion by automated system. False positive (FP), the number of false decision related to positive emotion by automated system. True negative (TN), the number of true decision related to negative emotion, and False negative (FN), the number of false decision related to negative emotion.

## Experimental results

### Channel selection

In this study, MLPNN was firstly used for channel selection. EEG recordings measured from 32 channels for every participant were evaluated separately and five EEG channels having the highest performance were dynamically determined. As we evaluate the results related to all participants, same channels provided the highest performances. The classification of EEG signals were achieved by a dynamic model in which the channels were selected for each participant. The dynamic selection process is given below.

**Fig. 6:**
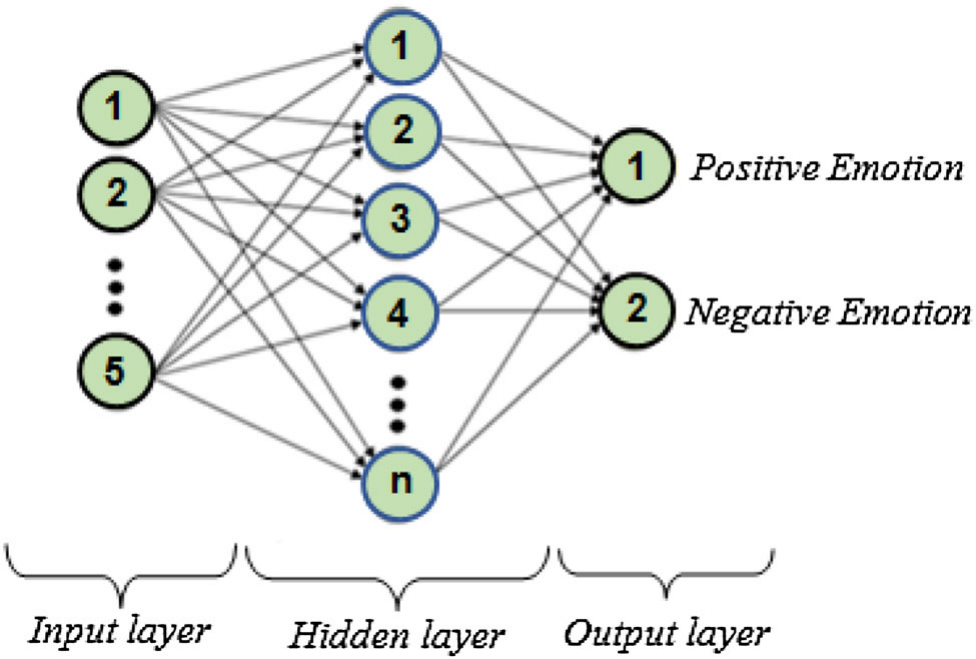
MLPNN structure: (5 × *n* × 2)

For every participant, feature vectors related to EEG segments consisting of positive and negative emotions were classified by a MLPNN. The feature vectors obtained by DWT with statistical calculation were used as input sets for MLPNN. The number of neurons in the input layer of MLPNN was five due to the size of feature vector. MLPNN output vectors were defined as [1 0] for positive emotion and [0 1] for negative emotion. Thus, the number of neurons in the output layer of network was two. Hence, the structure used in this study was (5 × *n* × 2), which is shown in Fig. 6, where n represents the number of neurons in the hidden layer. The number of neurons used in the hidden layer is separately determined for each participant. Each participant had 40 EEG segments (training and testing patterns) totally. Thirty EEG segments were randomly selected for network training stage, and the remaining 10 EEG segments selected for testing stage. In order to increase reliability of the classification results, the training and testing data were randomly changed four times.

Single hidden layer with 5 × *n* × 2 architecture was used in MLPNN architecture for determining five EEG channels having the best classification performances. Accuracy (Eq. 6) was taken as model success criteria for determining the channels. In training stage of MLPNN, the network parameters are learning coefficient 0.7 and momentum coefficient 0.9.

**Fig. 7:**
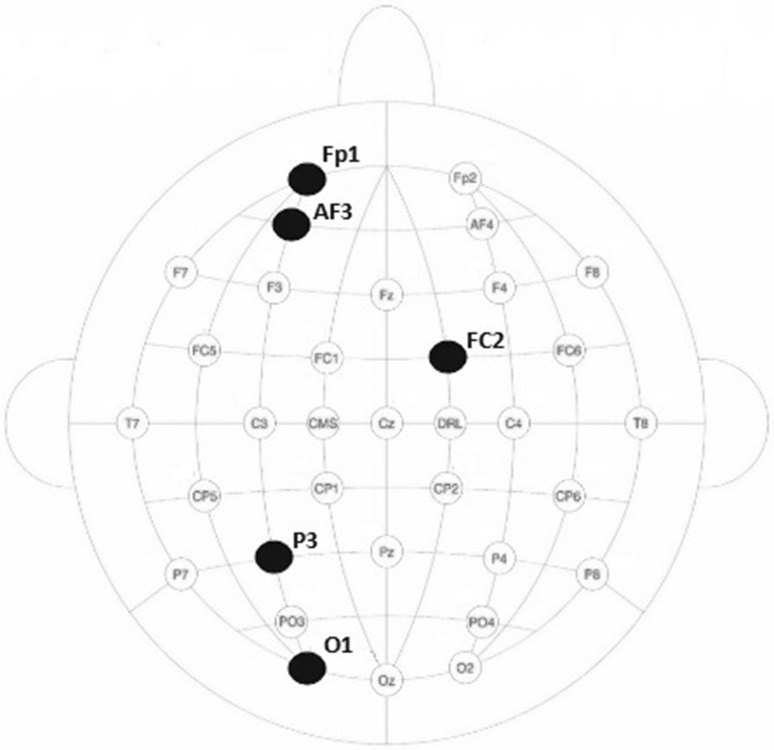
Positions of five EEG channels having the best classification performances

In this study, EEG signals recorded from 32 channels were examined, and five EEG channels having highest performance in emotion recognition were dynamically determined. It was generally observed that same channels except a few of them provided the highest performances. The main intention of determining the EEG channels is the simultaneous processing of EEG signals recorded from different regions of the brain. EEG signals recorded from different regions provide a more comprehensive and dynamic solution to the description of emotional state. At the end of the process, the classification results revealed that the channels having high performances were P3, FC2, AF3, O1 and Fp1 (Fig. 7). From this point of the study, those five channels were used instead of 32.

### Classification of emotions by using MLPNN

**Fig. 8:**
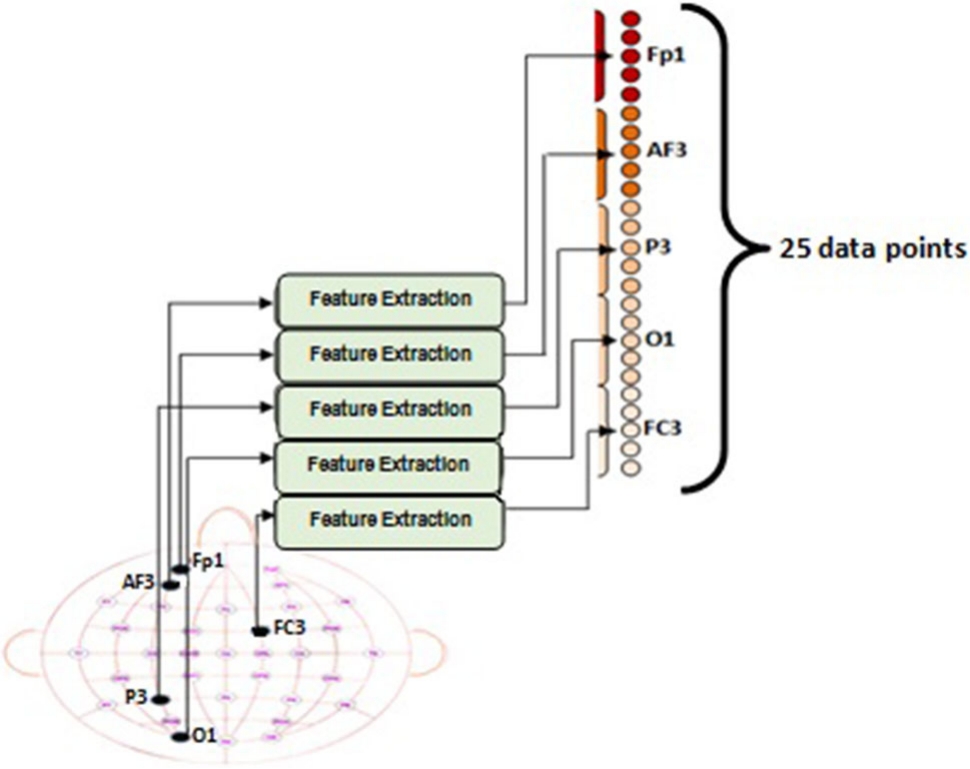
Formation and determination of final feature vectors

Final feature vectors were obtained by combining the features of EEG segments belonging to the selected channels (P3, FC2, AF3, O1 and Fp1). Thus, new feature vectors composed of 25 samples for every EEG segment related to positive and negative emotions were obtained. The formation procedure of final feature vectors with the selected five EEG channels is shown in Fig. 8. All procedures were applied separately for each participant.

The same procedure that was applied for channel selection was employed for the classification of emotions as well. MLPNN output vectors were defined as [1 0] for positive emotion and [0 1] for negative emotion. While training the network, 30 EEG segments are used and 10 EEG segments are used for testing. Single hidden layer with 25 × *n* × 2 architecture was used to classify EEG related with emotional states. The number of neurons used in the hidden layer is separately determined for each participant. In training stage, learning and momentum coefficients were 0.7 and 0.9, respectively.

The classification process was applied for each participant and results are shown in Table 2. According to SAM valence, two participants out of 22 lacked health assessment and for that reason classification process was not applied on them.

**Table 2:** Classification of emotions for each participant using MLPNN

Participants	Accuracy (%)	Specificity (%)	Sensitivity (%)
1	77.5	78.9	76.2
2	72.5	76.4	69.5
3	80	89.4	74.1
4	7	64.2	83.3
5	80	100	71.4
6	75	81.2	70.8
7	90	100	83.3
8	80	80	80
9	60	62.5	58.3
10	65	63.6	66.6
11	80	74.1	89.4
12	85	93.7	79.1
13	75	91.6	67.8
14	67.5	62.9	76.9
15	80	87.5	75
16	72.5	76.4	69.5
17	80	80	80
18	77.5	86.6	72
19	80	100	71.4
20	77.5	78.9	76.1
Average	77.14	92	76.75

As shown in Table 2, the percentages of accuracy, specificity, and sensitivity are in the range of [60 90], [62.5 100] and [58.3 89.4], respectively. To estimate the overall performance of the MLPNN model, statistical measures (accuracy, specificity, sensitivity) were averaged.

### Classification of emotion by using *k*NN algorithm

In this section, final feature vectors obtained from EEG segments were classified by using *k*NN algorithm. Since Euclidean distance measure method is frequently used in the literature [33], Euclidean distance measure was selected. Thirty EEG segments are used for the algorithm training and 10 EEG segment are used for testing. In order to increase reliability of the classification results, the training and testing data were randomly changed four times (fourfold cross-validation). Classification accuracies showed that *k* value offering the lowest error value varied for participants. From that point, *k* parameter is determined as 1 or 3 for each participant. The same procedure was applied for each participant and the classification performance in terms of statistical parameters is show in Table 3. As shown in the table, the percentages of accuracy, specificity, and sensitivity are in the range of [55 85], [53.5 100], and [63.1 85], respectively. To estimate the overall performance of the *k*NN model, statistical measures (accuracy, specificity, sensitivity) were averaged.

**Table 3:** Classification of emotions for each participant using *k*NN

Participants	Accuracy (%)	Specificity (%)	Sensitivity (%)
1	77.5	78.9	76.2
2	75	75	75
3	75	81.2	70.1
4	55	53.5	72.7
5	80	87.5	75
6	70	81.2	64.7
7	82.5	100	74
8	77.5	82.3	73.9
9	65	71.4	61.5
10	65	71.4	61.5
11	67.5	73.3	64
12	85	100	74
13	70	91.6	63.1
14	62.5	60.8	64.7
15	75	85.7	69.2
16	72.5	53.5	58.33
17	75	90.9	65.5
18	82.5	93.3	76
19	77.5	86.6	72
20	85	85	85
Average	72.92	90	74.37

The highest classification accuracy was obtained as 85%, and it was determined for participants 12 and 20, as seen in Table 3. For 20 participants evaluated, the average classification accuracy was determined as 72.92% using *k*NN algorithm.

**Fig. 9:**
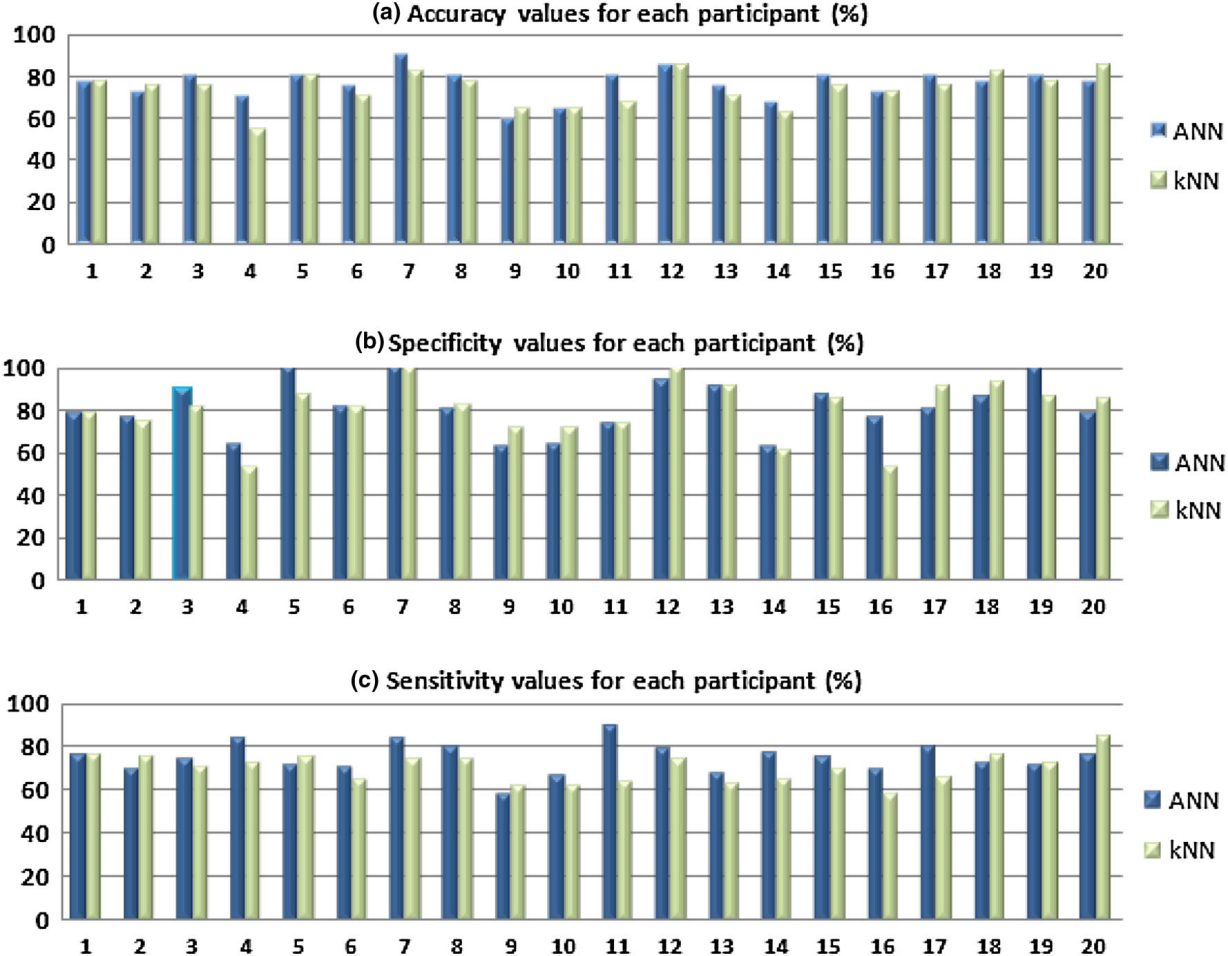
Comparison of classification performances for each participant in terms of, **a** accuracy, **b** specificity and, **c** sensitivity (*x*-*axis*—participants, *y-axis*—performances)

In order to compare the performance of two classifier algorithms, classification performances in terms of accuracy, sensitivity, and specificity are shown in Fig. 9.

## Results and discussion

The aim of this section of the paper is to examine, discuss, and compare the results obtained from the proposed methods with channel selection. The discussion of findings obtained from this study is presented below.

**Fig. 10:**
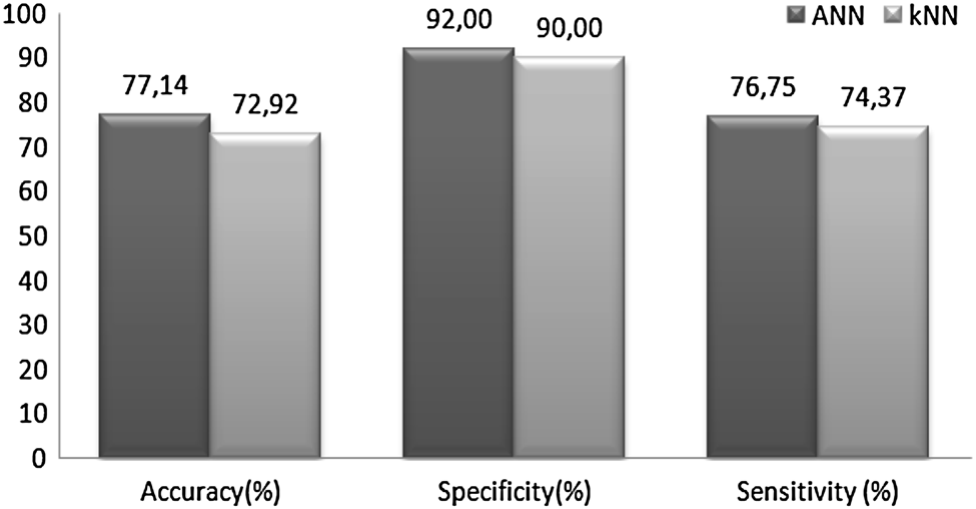
Overall performances of MLPNN and *k*NN classifiers
- It was concluded that MLPNN and *k*NN used in this study give good accuracy results for classification of emotions.

- Wavelet coefficients related to different emotions were obtained using DWT method. These wavelet coefficients were evaluated as feature vectors. The size of feature vectors was reduced by using five statistical parameters to get rid of the computing load.
- Thirty-two channels for every participant were evaluated separately, and five channels were determined. MLPNN (5 × 10 × 2) structure was used for channel selection. The results revealed that similar channels (P3, FC2, AF3, O1 and Fp1) had the highest performance for every participant. The channels having the highest performance were selected for classification of emotions. From this point of the study, five channels were used instead of 32. The results revealed that the channels accepted as brain regions had relation with emotions. The selected channels determined in this study were also compatible with the channels selected in other papers [21].
- Combined feature vectors of selected five channels were classified by using MLPNN and *k*NN methods. In reference to the literature, MLPNN is one of the most popular tools for EEG analysis. In this study, MLPNN was employed for determining the emotional state from the EEG signals. In this study, *k*NN algorithm was also used as a classifier for increasing the reliability of the results obtained by MLPNN. *k*NN is one of the most fundamental and simple classification methods. In many EEG applications, *k*NN algorithm is frequently used. The fact that the results produced by the two algorithms were close to each other. This matching support the process reliability.
- As shown in Table 2, the percentages of accuracy, specificity, and sensitivity of MLPNN are in the ranges of [60 90], [62.5 100], and [58.3 89.4], respectively. These values indicate that the proposed MLPNN model is successful, and the test results also show that the generalization ability of MLPNN is well. On the other hand, the percentages of accuracy, specificity, and sensitivity of *k*NN are in the ranges of [55 85], [53.5 100], and [63.1 85], respectively (Table 3). It was observed that both classifiers showed parallel performance for each participant. The best classification accuracy of MLPNN was obtained as 90% (specificity: 100% and sensitivity: 83.3%) for participant 7. On the other part, the best classification accuracy of *k*NN was obtained as 85% for participant 12 and 20.
- To estimate the overall performance of MLPNN and *k*NN classifiers, statistical measures (accuracy, specificity, sensitivity) were averaged. The comparison of averaged values for classification of emotions is shown in Fig. 10. As shown in the figure, the performance of MLPNN was higher than that of *k*NN. However, both methods can be accepted as successful.

The channel selection has opened the door to improve the performance of automatic detection for emotion recognition. Recently, Zhang et al [20] used two EEG channels (F3 and F4) for feature extraction. Bhardwaj et al. [21] used three EEG channels (Fp1, P3 and O1) in order to detect emotion. Murugappan et al. [18] extracted features from 64, 24 and 8 EEG channels, respectively. When these studies were reviewed, it can be observed that the main EEG channels and region of brain was considered for detection of emotions. However, in this study, all EEG channels were evaluated and considered separately and five EEG channels that offer the best classification performance were determined. Final feature vectors were obtained by combining the features of those EEG channels, and the classification performance was improved. In literature, same database was used by Atkinson and Campos [34] for emotion recognition. They obtained the classification accuracy as 73.14% in valence dimension. In this study, the better accuracy was obtained by applying channel selection. Further, the channel selection has been used to improve the other areas of EEG classification.

## Conclusion

The aim of this study was to classify EEG signals related to different emotions based on audiovisual stimuli with the preprocessing of channel selection.

In this study, the publicly available DEAP database related to emotional states were used. EEG signals recorded from 20 healthy subjects in Twente University were examined.

For classification of emotions, DWT was used as feature extraction, while MLPNN and *k*NN methods were used as classifiers.

In this study, dynamic channel selection was used for determining the channels having more relation with emotions. Five channels (P3, FC2, AF3, O1, and Fp1) having the highest performances selected successfully among the 32 channels with MLPNN.

The performance ranges of classification for emotions are compatible to both MLPNN and *k*NN. As shown from the results, the key points were feature and channel selections. It is considered that, with correct channels and features, the performance can be increased.

In literature, it can be seen that useful features can be obtained from the alpha, beta, and gamma bands as well as the tetra band in the detection of the emotional state from EEG signals. In future studies, the proposed model can be used for all sub-bands separately to identify the effectiveness of the bands in emotional activity. Furthermore, in order to increase the classification success, other physiological signals such as blood pressure, respiratory rate, body temperature, and GSR (galvanic skin response) can be used with EEG signals.
